# Beyond the AMPA receptor: Diverse roles of SynDIG/PRRT brain-specific transmembrane proteins at excitatory synapses

**DOI:** 10.1016/j.coph.2021.03.011

**Published:** 2021-05-05

**Authors:** Elva Dίaz

**Affiliations:** Department of Pharmacology, University of California Davis School of Medicine, 451 Health, Sciences Drive, Davis, CA 95616, USA

**Keywords:** SynDIG1, SynDIG4, PRRT1, PRRT2, AMPA receptor auxiliary factor, CP-AMPARs, Excitatory synapse, Synaptic plasticity, Paroxysmal kinesigenic dyskinesia, Palmitoylation

## Abstract

α-amino-3-hydroxy-5-methyl-4-isoxazolepropionic acid (AMPA)-type glutamate receptors (AMPARs) are responsible for fast excitatory transmission in the brain. Deficits in synaptic transmission underlie a variety of neurological and psychiatric disorders. However, drugs that target AMPARs are challenging to develop, given the central role played in neurotransmission. Targeting AMPAR auxiliary factors offers an innovative approach for achieving specificity without altering baseline synaptic transmission. This review focuses on the SynDIG/proline-rich transmembrane protein (PRRT) family of AMPAR-associated transmembrane proteins. Although these factors are related based on sequence similarity, the proteins have evolved diverse actions at excitatory synapses that are not limited to the traditional role ascribed to an AMPAR auxiliary factor. SynDIG4/PRRT1 acts as a typical AMPAR auxiliary protein, while PRRT2 functions at presynaptic sites to regulate synaptic vesicle dynamics and is the causative gene for neurological paroxysmal disorders in humans. SynDIG/PRRT proteins are members of a larger superfamily that also include antiviral proteins known to restrict fusion between host and viral membranes and share some interesting characteristics.

## Introduction

Aberrant excitatory neurotransmission underlies many neurological and psychiatric diseases, including Alzheimer’s disease, epilepsy, and schizophrenia. AMPA-type glutamate receptors (AMPARs) mediate the majority of fast excitatory neurotransmission. Activity-dependent changes in synaptic AMPAR levels are driven by long-term potentiation (LTP) and long-term depression (LTD) of synaptic strength, which are cellular mechanisms proposed to underlie learning and memory. The molecular mechanisms that regulate AMPAR trafficking and dynamics at excitatory synapses have been intensely studied for over two decades [1]. AMPARs consist of four core subunits (GluA1-4) with GluA1/A2 complexes accounting for ~80%, and GluA2/A3 largely the rest, of synaptic AMPARs under basal conditions in the hippocampus [2]. AMPAR postsynaptic localization depends on postsynaptic density (postsynaptic density protein of 95kDa (PSD-95) [3] and the interaction with TARPs (transmembrane AMPAR-associated regulatory proteins), established auxiliary subunits that promote surface expression and channel activity [4–6]. In addition, a number of structurally not related transmembrane AMPAR accessory protein families have been identified with distinct and overlapping functions [7,8; see Abdolah Nejat, this issue]. Accessory proteins that associate with receptor complexes represent promising targets for selective drug development [9]. A recent example is the development of a forebrain-selective TARPγ8-associated AMPAR antagonist for epilepsy [10].

This review focuses on one group of AMPAR accessory proteins called synapse differentiation–induced gene proteins (SynDIG) and proline-rich transmembrane proteins (PRRT) (Table 1). SynDIG/PRRT are highly conserved brain-specific type II transmembrane proteins with a single transmembrane domain and a second hydrophobic segment that does not span the membrane [11–13]. The SynDIG/PRRT proteins belong to a larger superfamily misnamed the “dispanins” because of the prediction of two transmembrane domains [14], which also includes the interferon-induced transmembrane proteins (IFITMs), molecules that restrict cellular infection by pathogenic viruses [15]. Members of the superfamily share a distinctive topology and lipid modification important for their function.

## An atypical AMPAR auxiliary factor: SynDIG1

**Figure 1:**
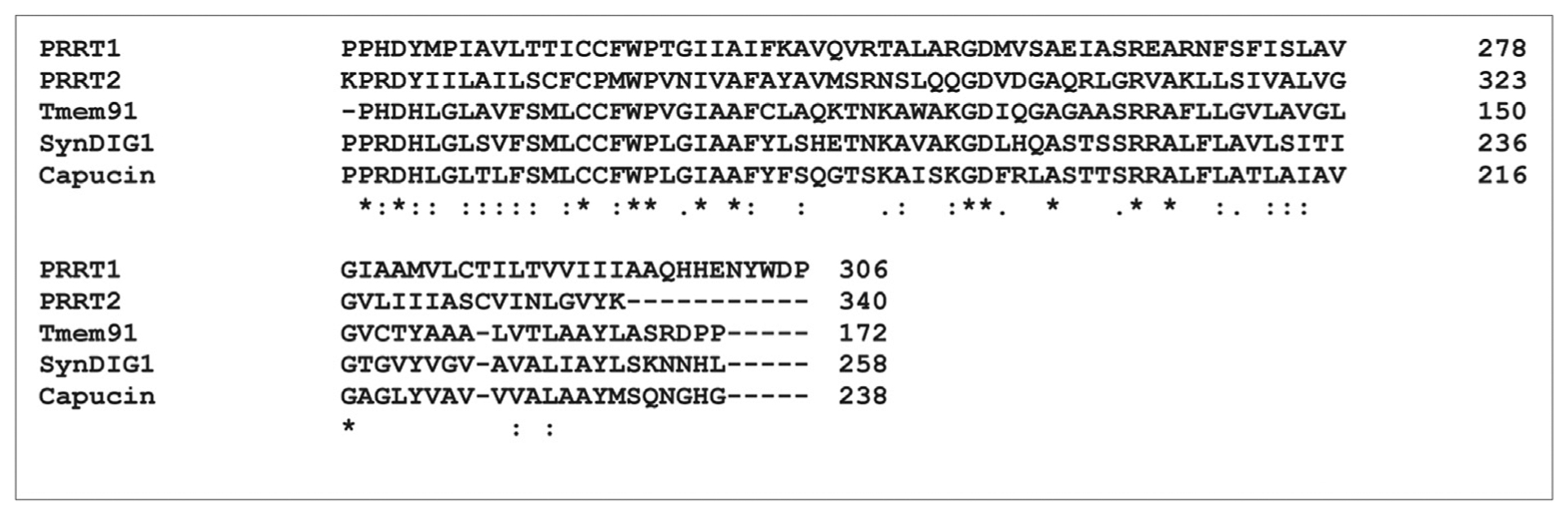
Sequence alignment of SynDIG and PRRT C-terminal regions. Clustal Omega (1.2.4) multiple sequence alignment of amino acids within the membrane-associated regions from SynDIG and PRRT proteins. GenBank accession numbers: PRRT1 (NP_085154.3); PRRT2 (NP_660282.2); Tmem91 (NP_001092291.1); SynDIG1 (NP_079169.1); Capucin (NP_001099049.1); Identical amino acids are indicated with an asterisk. Similar amino acids are indicated with a colon (strong similarity) or period (low similarity).

SynDIG1 was identified in an expression profiling screen as upregulated during synapse development in the cerebellum [16]. Overexpression or knockdown of SynDIG1 in dissociated rat hippocampal neurons increased or decreased, respectively, AMPAR synapse size and number by ~50%, with immunocytochemistry and electrophysiology [11], thereby establishing SynDIG1 as a central regulator of excitatory synapse development. Transgenic mice with a targeted mutation in SynDIG1 exhibit deficits in excitatory synapse maturation in the absence of presynaptic effects [17], consistent with a postsynaptic role. Surprisingly and in contrast to other AMPAR accessory proteins, SynDIG1 does not alter AMPAR surface expression or biophysical properties [18], suggesting that SynDIG1 does not function as a traditional auxiliary factor. Sequence similarity is observed among five gene products (Table 1), with the highest degree of similarity in the second half of the protein that includes the membrane-associated regions (Figure 1). Two of the related proteins are called PRRT1 and PRRT2 to reflect the abundance of proline residues in the intracellular N-terminal region. Tmem91 remains uncharacterized, and little is known about Capucin, which was identified based on its high expression in the caudate putamen of the dorsolateral striatum and is downregulated in rodent models of Huntington [19], suggesting a potential role in Huntington disease. However, neither Capucin deficiency nor overexpression altered the toxicity of a mutant Huntingtin fragment in vivo [20].

## An auxiliary factor for nonsynaptic AMPARs: SynDIG4/PRRT1

PRRT1, which was named SynDIG4 based on sequence similarity [11] and referred to here as SynDIG4/PRRT1, was identified in multiple independent proteomic studies of AMPAR complexes [21–25]. Surprisingly, however, SynDIG4/PRRT1 is not enriched in the PSD but instead colocalizes with GluA1-containing AMPARs at nonsynaptic sites [12]. Nonetheless, SynDIG4/PRRT1 alters AMPAR biophysical properties in a subunit-specific manner [26**], indicating a direct and specific interaction of SynDIG4/PRRT1 with GluA1-containing AMPARs. Remarkably, tetanus-induced LTP, which is dependent on GluA1, is abolished in acute hippocampal slices from SynDIG4/PRRT1 knockout (KO) mice while theta burst stimulation LTP, which is independent of GluA1, is not impaired [26**]. During LTP, GluA1-containing AMPARs are recruited from nearby reserve pools, including perisynaptic regions on the cell surface and intracellular compartments [27]. Indeed, extrasynaptic GluA1 and GluA2 density is reduced in SynDIG4/PRRT1 KO neurons [26**]. GluA1/2 heteromers constitute 95% of the surface extrasynaptic AMPAR pool under basal conditions [2], suggesting that SynDIG4/PRRT1 is required to maintain reserve pools of extrasynaptic GluA1/2 heteromers. Troyano-Rodriguez et al. demonstrated that GluA1 and GluA2 surface levels are reduced in SynDIG4/PRRT1 KO hippocampus without an effect on baseline neurotransmission [28**]. The authors further showed that GluA1 pS845 and pS831 are reduced or increased in hippocampal lysates from SynDIG4/PRRT1 KO mice, respectively [28**]. Phosphorylation of GluA1 on S845 by PKA augments its surface expression and AMPAR targeting to the reserve pool required for LTP [29–32]. Together, these data are consistent with a model in which SynDIG4/PRRT1 maintains reserve pools of GluA1-containing AMPARs outside of the PSD that are targeted to synapses during LTP. SynDIG4/PRRT1 association with AMPARs, however, is not altered with phosphorylation deficient or mimetic mutants [28**]; thus, the mechanism by which SynDIG4/PRRT1 establishes a reserve pool of extrasynaptic AMPARs is unclear.

Certain effects of SynDIG4/PRRT1 were selective for GluA1. For example, puncta size and intensity of both extrasynaptic and synaptic GluA1 (but not GluA2) were slightly reduced in SynDIG4/PRRT1 KO neurons [26**], indicating an additional role in regulating synaptic GluA1. Furthermore, SynDIG4/PRRT1 slows desensitization of GluA1 homomers but not GluA1/2 heteromers in Xenopus oocytes [26**]. Interestingly, calcium-permeable CP-AMPARs, which consist primarily of GluA1 homomers in hippocampus and are largely absent at PSDs under basal conditions [33], are transiently inserted into postsynaptic sites during certain forms of LTP and at certain ages [34–36]. For example, a minimal induction protocol (1 tetanus, 100 Hz, 1 sec) requires CP-AMPARs, and this protocol fails to induce LTP in SynDIG4/PRRT1 KO hippocampal slices [26**]. Similarly, NMDAR-dependent LTD also requires temporary postsynaptic insertion of CP-AMPARs that is dependent on GluA1 S845 phosphorylation by PKA [37]. Intriguingly, NMDA-dependent LTD is absent in SynDIG4/PRRT1 KO hippocampus [28**], suggesting a selective role for SynDIG4/PRRT1 in plasticity mechanisms that rely on CP-AMPARs. Indeed, accumulation of GluA1 homomers at perisynaptic sites requires S845 phosphorylation [38], in line with the reduction in GluA1 pS854 in SynDIG4/PRRT1 KO hippocampus [28**].

## An AMPAR auxiliary factor in the wrong place: Prrt2

Similar to SynDIG4/PRRT1, PRRT2 was identified in the same proteomic screens as a component of AMPAR complexes [21–24], suggesting a postsynaptic function. However, recent studies have demonstrated that PRRT2 instead functions at presynaptic sites to modulate synaptic vesicle fusion dynamics. The first hint at a presynaptic function was demonstrated by knockdown studies in which defects in synapse formation and neurotransmitter released were observed [39]. Furthermore, the authors demonstrated that PRRT2 interacts with synaptotagmin, a Ca2+ sensor, and SNAP25, a component of synaptic vesicle SNARE complexes [39]. Using a reconstituted in vitro fusion assay with purified proteins, Coleman et al. demonstrated that PRRT2 negatively regulates the docking and priming stage of synaptic vesicle exocytosis via direct interaction with the SNARE machinery with its N-terminal proline-rich region [40**].

Intriguingly, PRRT2 is the causative gene for a variety of neurological disorders that are paroxysmal in nature, including paroxysmal kinesigenic dyskinesia and benign familial infantile seizures [41–43]. Truncating variants are distributed throughout the molecule and are loss of function mutations because of nonsense-mediated decay of the messenger RNA. Some pathogenic missense mutations cluster in the C-terminus and disrupt localization to the plasma membrane [44], consistent with disease progression because of loss of protein at the cell surface. However, one C-terminal missense mutation (G305W) that is not subject to nonsense-mediated decay leads to a complete disruption of the SNARE-inhibitory function of PRRT2 [40**], implying that the C-terminus co-ordinates with the N-terminal region to modulate membrane fusion.

PRRT2-mutant rodent models recapitulate aspects of the disease. PRRT2 KO mice exhibit spontaneous paroxysmal episodes consistent with seizure and movement disorders [45,46*] as well as cognitive deficits in spatial learning [47*]. Inhibitory and excitatory neurotransmission are oppositely altered in PRRT2 KO hippocampal neurons [48**], suggesting that hyperexcitability might underlie the paroxysmal phenotypes associated with PRRT2 disease mutations. Similar phenotypic results were obtained with a PRRT2 KO truncated mutant rat model [49*]. In both rodent models, a disrupted balance between excitatory and inhibitory neurotransmission as indicated by increased ratio of mEPSC/mIPSC events is observed, which likely leads to abnormal neuronal hyperexcitability. Interestingly, GluA1 levels were increased in PRRT2 mutant rats, whereas the GABA receptor subunit GABRA1 was decreased [49*]; however, it is not clear if these are direct or indirect effects of PRRT2 deficiency. An open question is whether PRRT2, such as SynDIG4/PRRT1, alters biophysical properties of AMPARs and if postsynaptic effects of PRRT2 contribute to disease. Very low levels of PRRT2 are present in PSD fractions [39], consistent with at least a portion of the protein having a postsynaptic role.

**Figure 2:**
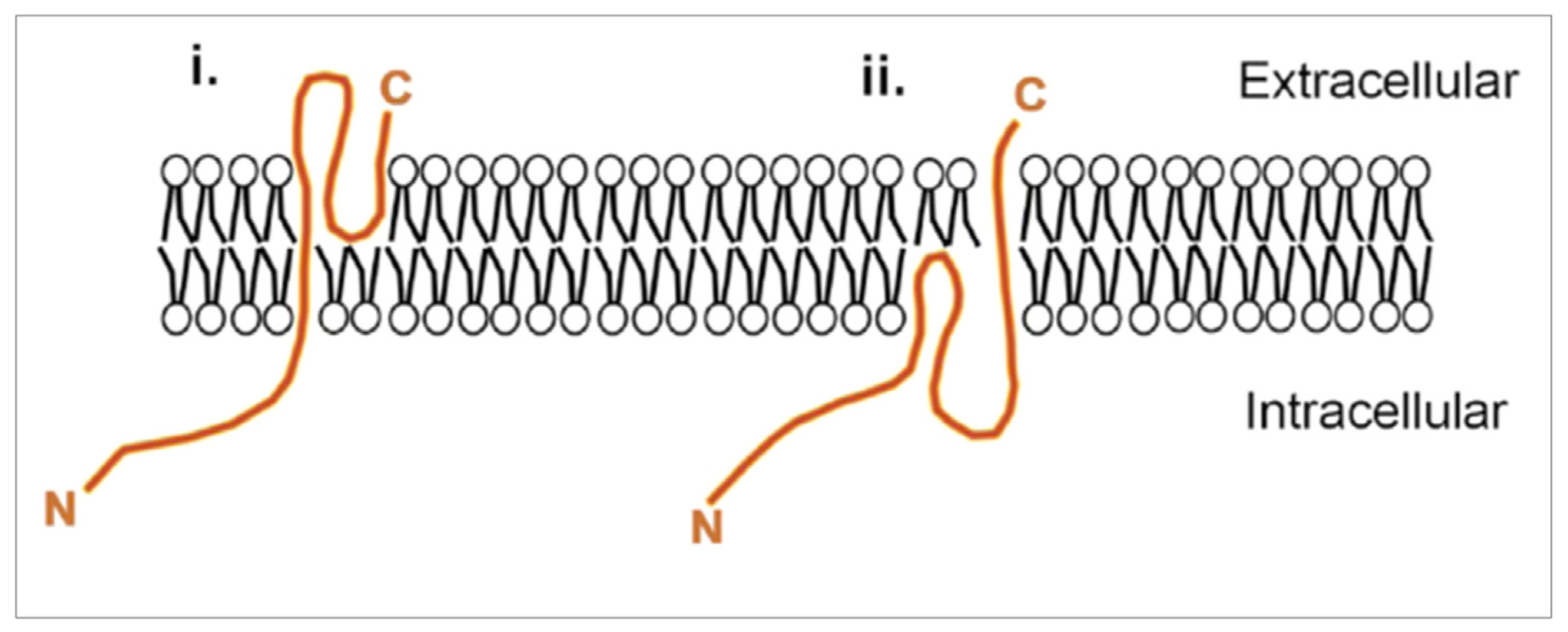
Topology of SynDIG and PRRT proteins. Schematic of possible models for SynDIG and PRRT topology based on epitope tagging and structural modeling. The loop region between the two predicted membrane segments could be either extracellular (1) as demonstrated for SynDIG1 or intracellular (2) as demonstrated for PRRT2. The topology for SynDIG4/PRRT1 has not been determined. Models are not to scale. Schematic reproduced from Ref. [12].

Common SynDIG and PRRT structural and posttranslational modifications Epitope tagging revealed that SynDIG1 is a type II transmembrane protein with a single membrane-spanning domain and a second hydrophobic segment that does not span the membrane [11]. The membrane-associated regions are important for AMPAR association and clustering within heterologous cells [11]. Subsequent structural modeling predicted that the second hydrophobic segment forms two intramembrane helices [50]. A similar topology has been verified for SynDIG4/PRRT1 [12] and PRRT2 [13], resulting in a large intracellular N-terminus and a short extracellular C-terminus (Figure 2).

**Figure 3:**
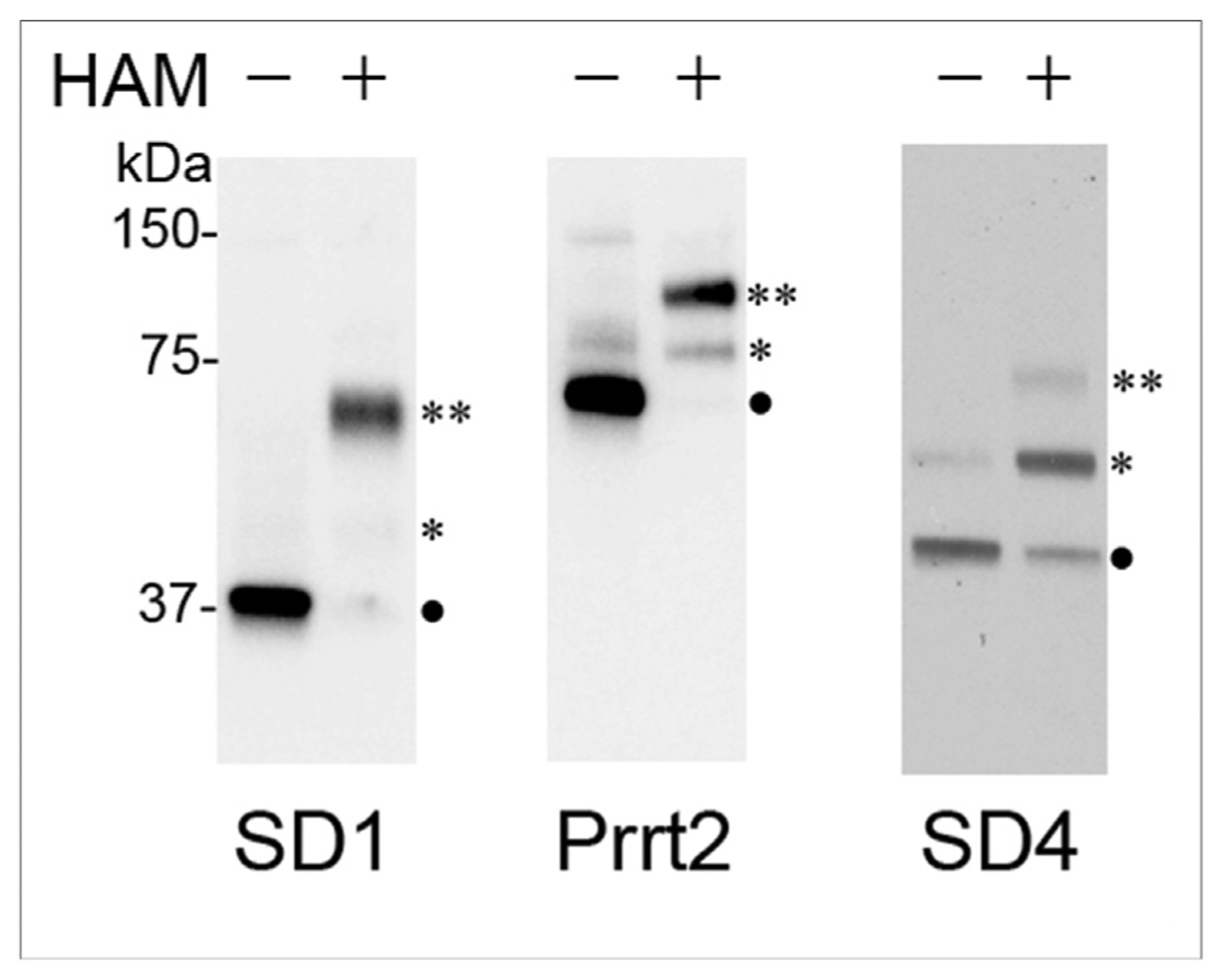
SynDIG and PRRT proteins are palmitoylated in brain. Detection of PEGylated proteins from postnatal day 18 mouse brain membrane lysates using the APEGS assay. Proteins were separated using a 10% SDS-PAGE, transferred to nitrocellulose and immunoblotted with antibodies against SynDIG1 (SD1), PRRT2, and SynDIG4/PRRT1 (SD4), Symbols indicate protein that is not palmitoylated (•), palmitoylated singly (*), or doubly (**), Figure reproduced from Ref. [52].

SynDIG1 is palmitoylated at two conserved juxta-transmembrane Cys residues (found in all SynDIG and PRRT proteins; Figure 1) in an activity-dependent manner to regulate stability, localization, and function [50]. Palmitoylation is a reversible posttranslational modification that influences membrane localization, trafficking, and protein–protein interactions [51]. Similar to SynDIG1, SynDIG4/PRRT1 and PRRT2 are also palmitoylated in brain lysates, as demonstrated with an innovative biochemical approach, the acyl–PEGyl exchange gel shift assay, to investigate the palmitoylation state of any protein of interest (Figure 3 [52*];). PRRT2 migrates at a higher molecular weight than its predicted size, in contrast to SynDIG1 and SynDIG4/PRRT1. A recent report demonstrated that PRRT2 undergoes activity-dependent cleavage to a 12 kDa C-terminal fragment that migrates at its predicted molecular weight [53], suggesting that the proline-rich N-terminus is responsible for its high apparent mobility.

## Insights from the dispanin superfamily and future directions

The SynDIG/PRRT proteins belong to a larger superfamily misnamed the “dispanins” because of the prediction of two transmembrane domains [14], which also includes the IFITMs, molecules that restrict cellular infection by pathogenic viruses by inhibiting fusion between the viral and host membranes [15,54,55]. The antiviral effects are dependent on oligomerization and palmitoylation [56]. In addition, IFITM3 contains an amphipathic helix before the transmembrane domain that is also important for its antiviral activity [57]. Although the precise mechanism is unclear, current models suggest that IFITMs alter membrane curvature and/or fluidity to inhibit fusion events. Evolutionarily, IFITMs and PRRT2 are more similar to each other than SynDIG1 and SynDIG4/PRRT1 [14] and thus might share a common mechanism. For example, G305W mutation in PRRT2 is implicated in PDK. Intriguingly, a recent preprint reports that the homologous site in IFITM3 (G95) is important for oligomerization and antiviral activity (https://doi.org/10.1101/2020.05.14.096891), further highlighting their similarities.

SynDIG4/PRRT1 shows equivalent pairwise sequence alignment to SynDIG1 (23.4% identity and 35.5% similarity) compared with PRRT2 (29.7% identity and 38.9% similarity); thus, SynDIG4/PRRT1 seems to represent the bridge between the SynDIGs and PRRTs, consistent with the divisions of the dispanin superfamily [14]. Thus, the protein should be referred to as “SynDIG4/PRRT1” to highlight this fact. SynDIG1 and SynDIG4/PRRT1 contain a conserved CCFWP sequence within the membrane-associated domain, whereas PRRT2 contains the sequence CFCPMWP (Figure 1). Perhaps, the AMPAR auxiliary factor function is a newly acquired function for SynDIG4/PRRT1 within the larger superfamily. The relationship between SynDIG1 and SynDIG4/PRRT1, and potentially PRRT2, at postsynaptic sites is unknown. In addition to the main well-characterized isoforms, other isoforms exist because of alternative splicing result in different proteins (Table 1), and functional characterization of any of these isoforms has yet to be reported.

Taken together, AMPAR accessory proteins, such as SynDIG and PRRT, family members represent promising targets for selective drug development [9], as demonstrated by the development of a forebrain-selective TARPγ8-associated AMPAR antagonist for epilepsy [10]. In particular, the SynDIG4/PRRT1 AMPAR auxiliary factor might reflect a potential target for interventions involved in memory impairments. Perhaps, boosting the activity of SynDIG4/PRRT1 might increase the reserve pool of extrasynaptic AMPARs to promote cognitive function in disorders associated with memory loss, such as Alzheimer’s disease. Although overexpression of SynDIG1 increases the number and strength of excitatory synapses in hippocampal neurons [11], the consequence of SynDIG4/PRRT1 overexpression has yet to be reported. Thus, modulation of AMPAR complexes via selective targeting of SynDIG4/PRRT1 represents an unexplored pharmaceutical area for future research.

## Figures and Tables

**Table 1:** SynDIG and PRRT proteins.

Name	Also known as	Isoforms	Functional effects
SynDIG1	C20orf39, DSPC2, IFITMD5, TMEM90B	Isoform 1 (258 aa) Isoform X1 (267 aa) Isoform X2 (253 aa) Isoform X3 (232 aa) Isoform X4 (206 aa)	Promotes synaptic targeting and AMPAR clustering
Capucin	SynDIG1-like, DSPC1, IFITMD4, TMEM90A, SynDIG2	Isoform 1 (238 aa)	Unknown
Tmem91	DSPC3, IFITMD6, SynDIG3	Isoform a (172 aa) Isoform b (139 aa) Isoform c (133 aa) Isoform d (127 aa) Isoform e (123 aa)	Unknown
PRRT1	C6orf31, DSPD1, IFITMD7, NG5, SynDIG4	Isoform 1 (306 aa) Isoform 2 (225 aa) Isoform 3 (153 aa)	Promotes AMPAR surface expression; Slows deactivation and desensitization kinetics
PRRT2	BFIC2, BFIS2, DSPB3, DYT10, EKD1, FICCA, ICCA, IFITMD1, PKC	Isoform 1 (340 aa) Isoform 2 (394 aa) Isoform 3 (299 aa) Isoform X1 (394 aa) Isoform X2 (346 aa) Isoform X3 (340 aa)	Regulates synaptic vesicle release
